# Bis(tribenzyl­ammonium) tetra­chloridoaurate(III) chloride

**DOI:** 10.1107/S1600536810002886

**Published:** 2010-01-30

**Authors:** Yousef Fazaeli, Vahid Amani, Mostafa M. Amini, Hamid Reza Khavasi

**Affiliations:** a Department of Chemistry, Shahid Beheshti University, G. C., Evin, Tehran 1983963113, Iran

## Abstract

In the title compound, (C_21_H_22_N)_2_[AuCl_4_]Cl, the Au^III^ atom adopts a square-planar coordination geometry defined by four chloride ions. In the crystal structure, inter­molecular N—H⋯Cl hydrogen bonds link the organic cations and the uncoordinated chloride ion.

## Related literature

For related structures, see: Calleja *et al.* (2001 ▶); Hasan *et al.* (1999 ▶); Hojjat Kashani *et al.* (2008 ▶); Jarvinen *et al.* (1988 ▶); Johnson & Steed (1998 ▶); Safari *et al.* (2009 ▶); Yıldırım *et al.* (2009*a* ▶,*b* ▶); Yap *et al.* (1995 ▶); Yousefi *et al.* (2007 ▶); Zeng *et al.* (1994 ▶); Zhang *et al.* (2006 ▶).
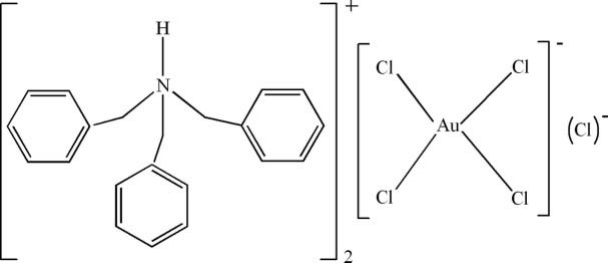


## Experimental

### 

#### Crystal data

(C_21_H_22_N)_2_[AuCl_4_]Cl
*M* *_r_* = 951.01
Triclinic, 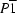

*a* = 11.135 (1) Å
*b* = 13.7920 (11) Å
*c* = 13.8417 (12) Å
α = 95.894 (7)°
β = 100.300 (7)°
γ = 95.222 (7)°
*V* = 2067.4 (3) Å^3^
*Z* = 2
Mo *K*α radiation
μ = 3.91 mm^−1^
*T* = 298 K
0.35 × 0.32 × 0.27 mm

#### Data collection

Stoe IPDS II diffractometer
Absorption correction: numerical (*X-RED*; Stoe & Cie, 2005 ▶) *T*_min_ = 0.280, *T*_max_ = 0.350
28425 measured reflections
12513 independent reflections
10542 reflections with *I* > 2σ(*I*)
*R*_int_ = 0.075

#### Refinement

*R*[*F* ^2^ > 2σ(*F* ^2^)] = 0.051
*wR*(*F* ^2^) = 0.144
*S* = 1.11
12513 reflections
451 parameters
H-atom parameters constrained
Δρ_max_ = 1.82 e Å^−3^
Δρ_min_ = −2.10 e Å^−3^

### 

Data collection: *X-AREA* (Stoe & Cie, 2005 ▶); cell refinement: *X-AREA*; data reduction: *X-RED* (Stoe & Cie, 2005 ▶); program(s) used to solve structure: *SHELXS97* (Sheldrick, 2008 ▶); program(s) used to refine structure: *SHELXL97* (Sheldrick, 2008 ▶); molecular graphics: *ORTEP-3* (Farrugia, 1997 ▶); software used to prepare material for publication: *WinGX* (Farrugia, 1999 ▶).

## Supplementary Material

Crystal structure: contains datablocks I, global. DOI: 10.1107/S1600536810002886/hb5291sup1.cif

Structure factors: contains datablocks I. DOI: 10.1107/S1600536810002886/hb5291Isup2.hkl

Additional supplementary materials:  crystallographic information; 3D view; checkCIF report

## Figures and Tables

**Table 1:** Selected bond lengths (Å)

Au1—Cl1	2.259 (2)
Au1—Cl2	2.2891 (15)
Au1—Cl3	2.2574 (17)
Au1—Cl4	2.2703 (15)

**Table 2:** Hydrogen-bond geometry (Å, °)

*D*—H⋯*A*	*D*—H	H⋯*A*	*D*⋯*A*	*D*—H⋯*A*
N1—H1*C*⋯Cl5	0.91	2.19	3.089 (5)	168
N2—H2⋯Cl5^i^	0.91	2.16	3.066 (4)	172

Symmetry code: (i) 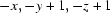.

**Fig. 1.**
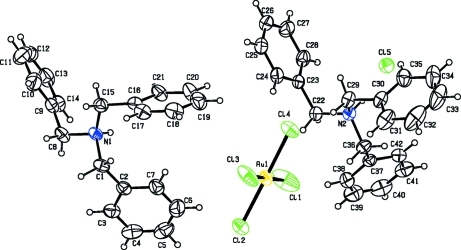
The molecular structure of (I). Displacement ellipsoids are drawn at the 30% probability level.

**Fig. 2.**
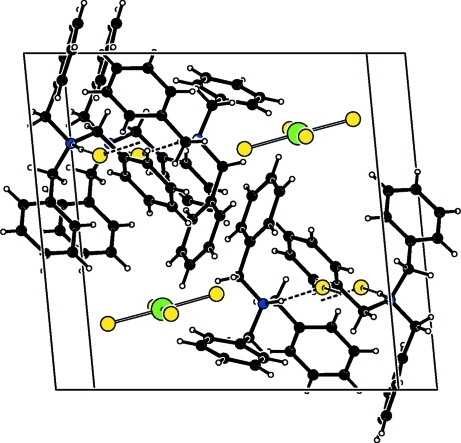
Unit-cell packing diagram for (I). Hydrogen bonds are shown as dashed lines.

## supplementary crystallographic information

### Comment

There are several proton transfer systems using tribenzylamine, with proton donor molecules, such as {(TBA)(DBA)[CuCl_4_]}, (II), (Zeng *et al.*, 1994), (TBA)[DCHSTO], (III), (Jarvine *et al.*, 1988) and {(TBA)_3_[PtCl_6_]Cl}, (IV), (Yousefi *et al.*, 2007) [where TBA is tribenzylammonium, DBA is dibenzylammonium and DCHSTO is 1,1,1,1,2,2,2,3,3,3-decacarbonyl-2,3-(µ-hydrido) -2,3-(µ-sulfonyl)-*triangulo*-tri-osmium] have been synthesized and characterized by single-crystal X-ray diffraction methods.

There are also several proton transfer systems using HAuCl_4_ with proton acceptor molecules, such as [EMI][AuCl_4_], (V) and [BMI]_2_[AuCl_4_].2H_2_O, (VI), (Hasan *et al.*, 1999), [H_2_bipy][AuCl_4_][Cl], (VII), (Zhang *et al.*, 2006), [H_7_O_3_][15-crown-5][AuCl_4_], (VIII) and [H_5_O_2_][benzo-15-crown-5]_2_[AuCl_4_], (IX), (Johnson & Steed, 1998), [H_5_O_2_]_2_[12-crown-4]_2_[AuCl_4_]_2_, (*X*), [H_3_O][18-crown-6][AuCl_4_], (XI) and [H_3_O] [4-nitrobenzo-18-crown-6][AuCl_4_], (XII), (Calleja *et al.*, 2001), [DPpy.H][AuCl_4_], (XIII), (Yap *et al.*, 1995), [H_2_DA18C6][AuCl_4_].2H2O, (XIV), (Hojjat Kashani *et al.*, 2008), [dafonium][dafone][AuCl_4_], (XV), (Safari *et al.*, 2009), [pz(py)_2_.H][AuCl_4_], (XVI), (Yıldırım, Akkurt, Safari *et al.*, 2009*a*), [Ph_2_Phen.H][AuCl_4_], (XVII), (Yıldırım, Akkurt, Safari, Abedi *et al.*, 2009*b*) [Where EMI is 1-ethyl-3-methylimidazolium, BMI is 1-butyl-3-methylimidazolium, H_2_bipy is 2,2'-bipyridinium, DPpy.H is 2,6-diphenylpyridinium, H_2_DA18C6 is 1,10-diazonia-18-crown-6, dafonium is 9-oxo-4,5-diazafluoren-4-ium, dafone is 4,5-diazafluoren-9-one, pz(py)_2_.H is 2-(3-pyridin-2-ylpyrazin-2-yl)pyridinium and Ph_2_Phen.H is 2,9-dimethyl-4,7-diphenyl-1,10- phenanthrolin-1-ium] have been synthesized and characterized by single-crystal X-ray diffraction methods. We report herein the synthesis and crystal structure of the title compound, (I).

The molecule of the title compound, (I), (Fig. 1), contains two independent protonated tribenzylammonium cations and [AuCl_4_]^-^ and Cl^-^ anions. The Au^III^ atom has a squareplanar environment defined by four Cl atoms. The bond lengths and angles, in cation, are in good agreement with the corresponding values in (II), (III) and (IV). In [AuCl_4_]^-^ anion, the Au—Cl bond lengths and angles (Table 1) are within normal range (*X*, XIII, XIV, XV and XVI).

In the crystal structure, intermolecular N—H···Cl hydrogen bonds (Table 2) result in the formation of a supramolecular structure (Fig. 2).

### Experimental

A solution of tribenzylamine (0.22 g, 0.74 mmol) in methanol (15 ml) was added to a solution of HAuCl_4_.3H_2_O, (0.29 g, 0.74 mmol) in acetonitrile (15 ml) and the resulting yellow solution was stirred for 30 min at 313 K. Then, it was left to evaporate slowly at room temperature. After five days, yellow blocks of (I) were isolated (yield 0.50 g; 71.1%; m.p. < 573 K).

### Refinement

All H atoms were positioned geometrically, with C—H = 0.93Å and constrained to ride on their parent atoms, with *U*_iso_(H) = 1.2*U*_eq_(C).

### Crystal data

**Table:**

(C_21_H_22_N)_2_[AuCl_4_]Cl	*Z* = 2
*M**_r_* = 951.01	*F*(000) = 948
Triclinic, *P*1	*D*_x_ = 1.528 Mg m^−^^3^
Hall symbol: -P 1	Mo *K*α radiation, λ = 0.71073 Å
*a* = 11.135 (1) Å	Cell parameters from 1237 reflections
*b* = 13.7920 (11) Å	θ = 1.9–30.6°
*c* = 13.8417 (12) Å	µ = 3.91 mm^−^^1^
α = 95.894 (7)°	*T* = 298 K
β = 100.300 (7)°	Block, yellow
γ = 95.222 (7)°	0.35 × 0.32 × 0.27 mm
*V* = 2067.4 (3) Å^3^	

### Data collection

**Table:**

Stoe IPDS II diffractometer	12513 independent reflections
Radiation source: fine-focus sealed tube	10542 reflections with *I* > 2σ(*I*)
graphite	*R*_int_ = 0.075
Detector resolution: 0.15 mm pixels mm^-1^	θ_max_ = 30.6°, θ_min_ = 1.9°
rotation method scans	*h* = −15→15
Absorption correction: numerical (*X-RED*; Stoe & Cie, 2005)	*k* = −19→19
*T*_min_ = 0.280, *T*_max_ = 0.350	*l* = −19→19
28425 measured reflections	

### Refinement

**Table:**

Refinement on *F*^2^	Primary atom site location: structure-invariant direct methods
Least-squares matrix: full	Secondary atom site location: difference Fourier map
*R*[*F*^2^ > 2σ(*F*^2^)] = 0.051	Hydrogen site location: inferred from neighbouring sites
*wR*(*F*^2^) = 0.144	H-atom parameters constrained
*S* = 1.11	*w* = 1/[σ^2^(*F*_o_^2^) + (0.0689*P*)^2^ + 1.4093*P*] where *P* = (*F*_o_^2^ + 2*F*_c_^2^)/3
12513 reflections	(Δ/σ)_max_ = 0.002
451 parameters	Δρ_max_ = 1.82 e Å^−^^3^
0 restraints	Δρ_min_ = −2.10 e Å^−^^3^

### Special details

**Table:**

Geometry. All e.s.d.'s (except the e.s.d. in the dihedral angle between two l.s. planes) are estimated using the full covariance matrix. The cell e.s.d.'s are taken into account individually in the estimation of e.s.d.'s in distances, angles and torsion angles; correlations between e.s.d.'s in cell parameters are only used when they are defined by crystal symmetry. An approximate (isotropic) treatment of cell e.s.d.'s is used for estimating e.s.d.'s involving l.s. planes.
Refinement. Refinement of *F*^2^ against ALL reflections. The weighted *R*-factor *wR* and goodness of fit *S* are based on *F*^2^, conventional *R*-factors *R* are based on *F*, with *F* set to zero for negative *F*^2^. The threshold expression of *F*^2^ > σ(*F*^2^) is used only for calculating *R*-factors(gt) *etc*. and is not relevant to the choice of reflections for refinement. *R*-factors based on *F*^2^ are statistically about twice as large as those based on *F*, and *R*- factors based on ALL data will be even larger.

### Fractional atomic coordinates and isotropic or equivalent isotropic displacement parameters (Å^2^)

**Table:**

	*x*	*y*	*z*	*U*_iso_*/*U*_eq_	
C1	0.3537 (5)	0.9891 (3)	0.3590 (4)	0.0603 (11)	
H1A	0.4326	0.9667	0.3824	0.072*	
H1B	0.3690	1.0542	0.3399	0.072*	
C2	0.2835 (4)	0.9955 (3)	0.4423 (4)	0.0568 (10)	
C3	0.2041 (5)	1.0661 (4)	0.4502 (4)	0.0688 (13)	
H3	0.1935	1.1098	0.4032	0.083*	
C4	0.1403 (6)	1.0724 (6)	0.5269 (6)	0.087 (2)	
H4	0.0861	1.1194	0.5309	0.104*	
C5	0.1563 (8)	1.0110 (7)	0.5954 (6)	0.098 (3)	
H5	0.1133	1.0161	0.6471	0.117*	
C6	0.2359 (8)	0.9397 (6)	0.5909 (5)	0.092 (2)	
H6	0.2465	0.8973	0.6391	0.110*	
C7	0.2996 (6)	0.9327 (4)	0.5134 (5)	0.0724 (14)	
H7	0.3534	0.8853	0.5095	0.087*	
C8	0.2330 (6)	0.9805 (4)	0.1885 (5)	0.0680 (13)	
H8A	0.1781	1.0218	0.2157	0.082*	
H8B	0.2997	1.0236	0.1742	0.082*	
C9	0.1643 (5)	0.9255 (3)	0.0930 (4)	0.0624 (11)	
C10	0.2214 (8)	0.9082 (6)	0.0137 (6)	0.091 (2)	
H10	0.3051	0.9275	0.0206	0.109*	
C11	0.1552 (12)	0.8619 (8)	−0.0770 (6)	0.116 (3)	
H11	0.1952	0.8477	−0.1294	0.140*	
C12	0.0306 (13)	0.8374 (7)	−0.0885 (7)	0.119 (4)	
H12	−0.0146	0.8087	−0.1494	0.142*	
C13	−0.0264 (8)	0.8554 (5)	−0.0104 (6)	0.092 (2)	
H13	−0.1104	0.8374	−0.0181	0.111*	
C14	0.0386 (6)	0.9001 (4)	0.0804 (5)	0.0710 (14)	
H14	−0.0019	0.9131	0.1327	0.085*	
C15	0.3678 (5)	0.8484 (4)	0.2333 (4)	0.0622 (11)	
H15A	0.4387	0.8843	0.2165	0.075*	
H15B	0.3234	0.8080	0.1740	0.075*	
C16	0.4110 (4)	0.7832 (3)	0.3102 (4)	0.0547 (9)	
C17	0.5296 (5)	0.7974 (4)	0.3657 (5)	0.0694 (13)	
H17	0.5858	0.8469	0.3540	0.083*	
C18	0.5642 (6)	0.7389 (5)	0.4377 (5)	0.0822 (18)	
H18	0.6435	0.7495	0.4749	0.099*	
C19	0.4823 (8)	0.6642 (6)	0.4556 (6)	0.092 (2)	
H19	0.5059	0.6247	0.5046	0.110*	
C20	0.3664 (7)	0.6496 (5)	0.4004 (6)	0.089 (2)	
H20	0.3111	0.5996	0.4123	0.107*	
C21	0.3291 (6)	0.7067 (4)	0.3276 (5)	0.0709 (14)	
H21	0.2500	0.6947	0.2901	0.085*	
C22	0.0653 (5)	0.4432 (4)	0.6633 (4)	0.0592 (10)	
H22A	−0.0206	0.4479	0.6655	0.071*	
H22B	0.1076	0.5092	0.6768	0.071*	
C23	0.0748 (5)	0.4012 (3)	0.5602 (3)	0.0533 (9)	
C24	0.1722 (5)	0.4335 (4)	0.5175 (4)	0.0630 (11)	
H24	0.2313	0.4826	0.5525	0.076*	
C25	0.1835 (6)	0.3944 (5)	0.4239 (4)	0.0707 (14)	
H25	0.2504	0.4159	0.3969	0.085*	
C26	0.0949 (7)	0.3235 (5)	0.3713 (4)	0.0771 (16)	
H26	0.1019	0.2967	0.3083	0.093*	
C27	−0.0033 (7)	0.2920 (5)	0.4106 (4)	0.0820 (18)	
H27	−0.0638	0.2449	0.3739	0.098*	
C28	−0.0135 (5)	0.3306 (5)	0.5063 (4)	0.0684 (13)	
H28	−0.0801	0.3083	0.5332	0.082*	
C29	0.2478 (4)	0.3628 (4)	0.7359 (4)	0.0606 (11)	
H29A	0.2452	0.3257	0.6719	0.073*	
H29B	0.2985	0.4244	0.7384	0.073*	
C30	0.3066 (4)	0.3067 (5)	0.8150 (4)	0.0605 (11)	
C31	0.4046 (6)	0.3513 (7)	0.8857 (5)	0.093 (2)	
H31	0.4313	0.4173	0.8865	0.112*	
C32	0.4632 (8)	0.2975 (12)	0.9557 (7)	0.132 (4)	
H32	0.5293	0.3277	1.0034	0.159*	
C33	0.4255 (11)	0.2024 (12)	0.9551 (7)	0.134 (5)	
H33	0.4650	0.1674	1.0029	0.160*	
C34	0.3298 (10)	0.1564 (8)	0.8852 (8)	0.115 (3)	
H34	0.3051	0.0900	0.8848	0.138*	
C35	0.2689 (6)	0.2088 (5)	0.8143 (5)	0.0784 (16)	
H35	0.2032	0.1778	0.7668	0.094*	
C36	0.1151 (4)	0.4354 (4)	0.8449 (3)	0.0590 (11)	
H36A	0.1616	0.4997	0.8530	0.071*	
H36B	0.1556	0.3983	0.8945	0.071*	
C37	−0.0122 (4)	0.4478 (3)	0.8636 (3)	0.0525 (9)	
C38	−0.0641 (7)	0.5326 (5)	0.8472 (5)	0.0783 (16)	
H38	−0.0220	0.5826	0.8221	0.094*	
C39	−0.1807 (8)	0.5434 (7)	0.8686 (6)	0.099 (3)	
H39	−0.2164	0.6003	0.8559	0.118*	
C40	−0.2417 (7)	0.4738 (8)	0.9066 (6)	0.093 (2)	
H40	−0.3196	0.4818	0.9193	0.112*	
C41	−0.1895 (6)	0.3906 (6)	0.9269 (5)	0.084 (2)	
H41	−0.2310	0.3430	0.9555	0.101*	
C42	−0.0750 (5)	0.3766 (4)	0.9053 (4)	0.0644 (12)	
H42	−0.0403	0.3195	0.9187	0.077*	
N1	0.2864 (4)	0.9198 (3)	0.2678 (3)	0.0530 (8)	
H1C	0.2225	0.8847	0.2858	0.064*	
N2	0.1179 (3)	0.3846 (3)	0.7443 (3)	0.0480 (7)	
H2	0.0700	0.3262	0.7363	0.058*	
Cl1	0.27943 (18)	0.67440 (18)	0.7747 (3)	0.1484 (13)	
Cl2	0.45070 (15)	0.88061 (11)	0.80407 (14)	0.0772 (4)	
Cl3	0.67313 (17)	0.76296 (12)	0.7524 (2)	0.1114 (8)	
Cl4	0.49931 (17)	0.56099 (11)	0.71556 (16)	0.0871 (5)	
Cl5	0.04611 (12)	0.81169 (9)	0.30405 (10)	0.0636 (3)	
Au1	0.475074 (16)	0.719913 (12)	0.760740 (14)	0.05584 (7)	

### Atomic displacement parameters (Å^2^)

**Table:**

	*U*^11^	*U*^22^	*U*^33^	*U*^12^	*U*^13^	*U*^23^
C1	0.053 (2)	0.046 (2)	0.075 (3)	−0.0076 (18)	0.007 (2)	−0.006 (2)
C2	0.052 (2)	0.047 (2)	0.064 (3)	−0.0038 (17)	−0.0008 (19)	−0.0004 (18)
C3	0.069 (3)	0.062 (3)	0.070 (3)	0.011 (2)	0.002 (2)	−0.004 (2)
C4	0.068 (3)	0.096 (5)	0.087 (4)	0.005 (3)	0.011 (3)	−0.025 (4)
C5	0.090 (5)	0.121 (7)	0.070 (4)	−0.019 (5)	0.016 (4)	−0.017 (4)
C6	0.107 (5)	0.087 (4)	0.068 (4)	−0.019 (4)	−0.005 (4)	0.012 (3)
C7	0.078 (3)	0.059 (3)	0.072 (3)	0.001 (2)	−0.005 (3)	0.009 (2)
C8	0.071 (3)	0.044 (2)	0.086 (4)	0.000 (2)	0.007 (3)	0.011 (2)
C9	0.071 (3)	0.045 (2)	0.073 (3)	0.007 (2)	0.011 (2)	0.015 (2)
C10	0.098 (5)	0.098 (5)	0.089 (5)	0.027 (4)	0.032 (4)	0.027 (4)
C11	0.157 (9)	0.136 (8)	0.067 (4)	0.067 (7)	0.025 (5)	0.014 (4)
C12	0.178 (11)	0.085 (5)	0.075 (5)	0.029 (6)	−0.022 (6)	−0.004 (4)
C13	0.099 (5)	0.073 (4)	0.090 (5)	−0.007 (3)	−0.021 (4)	0.022 (3)
C14	0.074 (3)	0.062 (3)	0.076 (3)	0.004 (2)	0.006 (3)	0.017 (2)
C15	0.065 (3)	0.056 (2)	0.068 (3)	0.009 (2)	0.021 (2)	0.005 (2)
C16	0.054 (2)	0.053 (2)	0.056 (2)	0.0082 (18)	0.0115 (19)	−0.0006 (18)
C17	0.053 (2)	0.066 (3)	0.090 (4)	0.008 (2)	0.017 (3)	0.004 (3)
C18	0.067 (3)	0.083 (4)	0.088 (4)	0.018 (3)	−0.008 (3)	0.000 (3)
C19	0.103 (5)	0.076 (4)	0.096 (5)	0.028 (4)	0.004 (4)	0.021 (3)
C20	0.088 (4)	0.061 (3)	0.116 (6)	0.006 (3)	0.006 (4)	0.029 (3)
C21	0.063 (3)	0.052 (3)	0.091 (4)	0.001 (2)	−0.003 (3)	0.011 (2)
C22	0.070 (3)	0.056 (2)	0.052 (2)	0.012 (2)	0.008 (2)	0.0113 (18)
C23	0.061 (2)	0.052 (2)	0.046 (2)	0.0107 (18)	0.0032 (18)	0.0116 (16)
C24	0.063 (3)	0.063 (3)	0.061 (3)	−0.002 (2)	0.006 (2)	0.018 (2)
C25	0.079 (4)	0.072 (3)	0.065 (3)	0.010 (3)	0.017 (3)	0.023 (3)
C26	0.107 (5)	0.075 (3)	0.052 (3)	0.020 (3)	0.013 (3)	0.013 (2)
C27	0.096 (4)	0.084 (4)	0.054 (3)	−0.008 (3)	−0.006 (3)	0.005 (3)
C28	0.065 (3)	0.078 (3)	0.056 (3)	−0.007 (2)	−0.001 (2)	0.016 (2)
C29	0.047 (2)	0.082 (3)	0.055 (2)	0.008 (2)	0.0089 (19)	0.019 (2)
C30	0.043 (2)	0.090 (4)	0.050 (2)	0.012 (2)	0.0066 (17)	0.017 (2)
C31	0.058 (3)	0.143 (7)	0.071 (4)	0.003 (4)	−0.005 (3)	0.016 (4)
C32	0.074 (5)	0.246 (15)	0.072 (4)	0.035 (7)	−0.014 (4)	0.032 (7)
C33	0.121 (8)	0.220 (13)	0.090 (6)	0.097 (9)	0.026 (5)	0.079 (8)
C34	0.123 (7)	0.134 (8)	0.112 (6)	0.049 (6)	0.040 (6)	0.068 (6)
C35	0.076 (4)	0.083 (4)	0.081 (4)	0.022 (3)	0.010 (3)	0.027 (3)
C36	0.054 (2)	0.068 (3)	0.047 (2)	−0.001 (2)	−0.0006 (18)	−0.0025 (19)
C37	0.059 (2)	0.053 (2)	0.0426 (18)	0.0084 (18)	0.0023 (17)	0.0007 (16)
C38	0.101 (5)	0.068 (3)	0.071 (3)	0.026 (3)	0.018 (3)	0.018 (3)
C39	0.106 (6)	0.112 (6)	0.081 (4)	0.068 (5)	0.002 (4)	0.007 (4)
C40	0.062 (3)	0.131 (7)	0.079 (4)	0.025 (4)	0.002 (3)	−0.016 (4)
C41	0.076 (4)	0.096 (5)	0.076 (4)	−0.017 (3)	0.027 (3)	−0.014 (3)
C42	0.075 (3)	0.056 (3)	0.060 (3)	0.006 (2)	0.014 (2)	−0.001 (2)
N1	0.0501 (18)	0.0395 (16)	0.067 (2)	−0.0057 (13)	0.0113 (16)	0.0031 (15)
N2	0.0433 (16)	0.0518 (18)	0.0465 (17)	0.0007 (13)	0.0054 (13)	0.0048 (14)
Cl1	0.0593 (9)	0.0925 (14)	0.282 (4)	−0.0136 (9)	0.0373 (15)	−0.0173 (19)
Cl2	0.0738 (8)	0.0579 (7)	0.0990 (10)	0.0136 (6)	0.0098 (7)	0.0116 (6)
Cl3	0.0754 (9)	0.0559 (7)	0.217 (3)	0.0043 (7)	0.0601 (13)	0.0278 (11)
Cl4	0.0884 (10)	0.0512 (6)	0.1191 (13)	0.0014 (6)	0.0184 (9)	0.0055 (7)
Cl5	0.0573 (6)	0.0615 (6)	0.0690 (7)	−0.0125 (5)	0.0167 (5)	0.0028 (5)
Au1	0.05173 (10)	0.04902 (10)	0.06372 (12)	0.00099 (6)	0.00227 (7)	0.01189 (7)

### Geometric parameters (Å, °)

**Table:**

C1—C2	1.504 (8)	C22—H22B	0.9700
C1—N1	1.533 (6)	C23—C28	1.373 (7)
C1—H1A	0.9700	C23—C24	1.386 (7)
C1—H1B	0.9700	C24—C25	1.382 (8)
C2—C7	1.376 (8)	C24—H24	0.9300
C2—C3	1.381 (7)	C25—C26	1.371 (9)
C3—C4	1.379 (10)	C25—H25	0.9300
C3—H3	0.9300	C26—C27	1.362 (10)
C4—C5	1.334 (13)	C26—H26	0.9300
C4—H4	0.9300	C27—C28	1.403 (9)
C5—C6	1.386 (13)	C27—H27	0.9300
C5—H5	0.9300	C28—H28	0.9300
C6—C7	1.388 (11)	C29—C30	1.496 (7)
C6—H6	0.9300	C29—N2	1.525 (6)
C7—H7	0.9300	C29—H29A	0.9700
C8—C9	1.496 (8)	C29—H29B	0.9700
C8—N1	1.518 (7)	C30—C35	1.375 (9)
C8—H8A	0.9700	C30—C31	1.378 (8)
C8—H8B	0.9700	C31—C32	1.388 (13)
C9—C10	1.374 (9)	C31—H31	0.9300
C9—C14	1.387 (8)	C32—C33	1.339 (18)
C10—C11	1.394 (13)	C32—H32	0.9300
C10—H10	0.9300	C33—C34	1.363 (16)
C11—C12	1.375 (16)	C33—H33	0.9300
C11—H11	0.9300	C34—C35	1.393 (10)
C12—C13	1.361 (14)	C34—H34	0.9300
C12—H12	0.9300	C35—H35	0.9300
C13—C14	1.384 (9)	C36—N2	1.501 (6)
C13—H13	0.9300	C36—C37	1.506 (7)
C14—H14	0.9300	C36—H36A	0.9700
C15—N1	1.497 (6)	C36—H36B	0.9700
C15—C16	1.505 (7)	C37—C38	1.373 (8)
C15—H15A	0.9700	C37—C42	1.387 (7)
C15—H15B	0.9700	C38—C39	1.400 (11)
C16—C17	1.390 (7)	C38—H38	0.9300
C16—C21	1.399 (8)	C39—C40	1.332 (13)
C17—C18	1.369 (10)	C39—H39	0.9300
C17—H17	0.9300	C40—C41	1.364 (12)
C18—C19	1.385 (11)	C40—H40	0.9300
C18—H18	0.9300	C41—C42	1.386 (9)
C19—C20	1.364 (11)	C41—H41	0.9300
C19—H19	0.9300	C42—H42	0.9300
C20—C21	1.371 (9)	N1—H1C	0.9100
C20—H20	0.9300	N2—H2	0.9100
C21—H21	0.9300	Au1—Cl1	2.259 (2)
C22—C23	1.510 (7)	Au1—Cl2	2.2891 (15)
C22—N2	1.514 (6)	Au1—Cl3	2.2574 (17)
C22—H22A	0.9700	Au1—Cl4	2.2703 (15)
			
C2—C1—N1	113.4 (4)	C25—C24—C23	121.5 (5)
C2—C1—H1A	108.9	C25—C24—H24	119.2
N1—C1—H1A	108.9	C23—C24—H24	119.2
C2—C1—H1B	108.9	C26—C25—C24	119.3 (6)
N1—C1—H1B	108.9	C26—C25—H25	120.4
H1A—C1—H1B	107.7	C24—C25—H25	120.4
C7—C2—C3	118.8 (6)	C27—C26—C25	120.4 (6)
C7—C2—C1	120.5 (5)	C27—C26—H26	119.8
C3—C2—C1	120.7 (5)	C25—C26—H26	119.8
C4—C3—C2	120.7 (7)	C26—C27—C28	120.2 (6)
C4—C3—H3	119.7	C26—C27—H27	119.9
C2—C3—H3	119.7	C28—C27—H27	119.9
C5—C4—C3	120.1 (7)	C23—C28—C27	120.1 (6)
C5—C4—H4	120.0	C23—C28—H28	120.0
C3—C4—H4	120.0	C27—C28—H28	120.0
C4—C5—C6	121.2 (8)	C30—C29—N2	113.9 (4)
C4—C5—H5	119.4	C30—C29—H29A	108.8
C6—C5—H5	119.4	N2—C29—H29A	108.8
C5—C6—C7	118.9 (7)	C30—C29—H29B	108.8
C5—C6—H6	120.6	N2—C29—H29B	108.8
C7—C6—H6	120.6	H29A—C29—H29B	107.7
C2—C7—C6	120.3 (6)	C35—C30—C31	119.3 (6)
C2—C7—H7	119.8	C35—C30—C29	120.6 (5)
C6—C7—H7	119.8	C31—C30—C29	120.0 (6)
C9—C8—N1	116.9 (4)	C30—C31—C32	119.8 (10)
C9—C8—H8A	108.1	C30—C31—H31	120.1
N1—C8—H8A	108.1	C32—C31—H31	120.1
C9—C8—H8B	108.1	C33—C32—C31	120.5 (9)
N1—C8—H8B	108.1	C33—C32—H32	119.7
H8A—C8—H8B	107.3	C31—C32—H32	119.7
C10—C9—C14	119.0 (6)	C32—C33—C34	120.7 (8)
C10—C9—C8	120.9 (6)	C32—C33—H33	119.6
C14—C9—C8	119.8 (6)	C34—C33—H33	119.6
C9—C10—C11	120.6 (8)	C33—C34—C35	119.9 (10)
C9—C10—H10	119.7	C33—C34—H34	120.0
C11—C10—H10	119.7	C35—C34—H34	120.0
C12—C11—C10	119.6 (8)	C30—C35—C34	119.7 (8)
C12—C11—H11	120.2	C30—C35—H35	120.2
C10—C11—H11	120.2	C34—C35—H35	120.2
C13—C12—C11	119.7 (8)	N2—C36—C37	114.2 (4)
C13—C12—H12	120.1	N2—C36—H36A	108.7
C11—C12—H12	120.1	C37—C36—H36A	108.7
C12—C13—C14	121.1 (8)	N2—C36—H36B	108.7
C12—C13—H13	119.4	C37—C36—H36B	108.7
C14—C13—H13	119.4	H36A—C36—H36B	107.6
C13—C14—C9	119.8 (7)	C38—C37—C42	118.7 (5)
C13—C14—H14	120.1	C38—C37—C36	120.9 (5)
C9—C14—H14	120.1	C42—C37—C36	120.2 (5)
N1—C15—C16	112.6 (4)	C37—C38—C39	119.6 (7)
N1—C15—H15A	109.1	C37—C38—H38	120.2
C16—C15—H15A	109.1	C39—C38—H38	120.2
N1—C15—H15B	109.1	C40—C39—C38	121.3 (7)
C16—C15—H15B	109.1	C40—C39—H39	119.3
H15A—C15—H15B	107.8	C38—C39—H39	119.3
C17—C16—C21	118.8 (5)	C39—C40—C41	119.8 (7)
C17—C16—C15	122.0 (5)	C39—C40—H40	120.1
C21—C16—C15	119.2 (5)	C41—C40—H40	120.1
C18—C17—C16	120.3 (6)	C40—C41—C42	120.5 (7)
C18—C17—H17	119.8	C40—C41—H41	119.8
C16—C17—H17	119.8	C42—C41—H41	119.8
C17—C18—C19	120.7 (6)	C41—C42—C37	120.0 (6)
C17—C18—H18	119.6	C41—C42—H42	120.0
C19—C18—H18	119.6	C37—C42—H42	120.0
C20—C19—C18	118.9 (7)	C15—N1—C8	113.5 (4)
C20—C19—H19	120.5	C15—N1—C1	111.6 (4)
C18—C19—H19	120.5	C8—N1—C1	108.9 (4)
C19—C20—C21	121.8 (7)	C15—N1—H1C	107.5
C19—C20—H20	119.1	C8—N1—H1C	107.5
C21—C20—H20	119.1	C1—N1—H1C	107.5
C20—C21—C16	119.5 (6)	C36—N2—C22	111.4 (4)
C20—C21—H21	120.3	C36—N2—C29	111.1 (4)
C16—C21—H21	120.3	C22—N2—C29	111.0 (4)
C23—C22—N2	114.7 (4)	C36—N2—H2	107.7
C23—C22—H22A	108.6	C22—N2—H2	107.7
N2—C22—H22A	108.6	C29—N2—H2	107.7
C23—C22—H22B	108.6	Cl3—Au1—Cl1	177.75 (11)
N2—C22—H22B	108.6	Cl3—Au1—Cl4	89.38 (7)
H22A—C22—H22B	107.6	Cl1—Au1—Cl4	90.02 (8)
C28—C23—C24	118.5 (5)	Cl3—Au1—Cl2	90.38 (6)
C28—C23—C22	120.7 (5)	Cl1—Au1—Cl2	90.25 (8)
C24—C23—C22	120.8 (5)	Cl4—Au1—Cl2	179.20 (6)
			
N1—C1—C2—C7	90.4 (6)	C25—C26—C27—C28	−1.3 (11)
N1—C1—C2—C3	−91.2 (5)	C24—C23—C28—C27	0.7 (9)
C7—C2—C3—C4	−1.2 (8)	C22—C23—C28—C27	−179.9 (6)
C1—C2—C3—C4	−179.6 (5)	C26—C27—C28—C23	0.8 (10)
C2—C3—C4—C5	1.0 (9)	N2—C29—C30—C35	72.7 (7)
C3—C4—C5—C6	−0.4 (11)	N2—C29—C30—C31	−111.4 (6)
C4—C5—C6—C7	−0.1 (11)	C35—C30—C31—C32	−0.5 (11)
C3—C2—C7—C6	0.7 (8)	C29—C30—C31—C32	−176.4 (7)
C1—C2—C7—C6	179.1 (5)	C30—C31—C32—C33	0.0 (15)
C5—C6—C7—C2	−0.1 (10)	C31—C32—C33—C34	0.8 (17)
N1—C8—C9—C10	−95.6 (7)	C32—C33—C34—C35	−1.0 (16)
N1—C8—C9—C14	91.0 (6)	C31—C30—C35—C34	0.3 (10)
C14—C9—C10—C11	−2.8 (10)	C29—C30—C35—C34	176.1 (6)
C8—C9—C10—C11	−176.2 (7)	C33—C34—C35—C30	0.5 (13)
C9—C10—C11—C12	3.0 (14)	N2—C36—C37—C38	−94.3 (6)
C10—C11—C12—C13	−2.3 (15)	N2—C36—C37—C42	90.9 (6)
C11—C12—C13—C14	1.5 (13)	C42—C37—C38—C39	−2.8 (9)
C12—C13—C14—C9	−1.3 (10)	C36—C37—C38—C39	−177.8 (6)
C10—C9—C14—C13	1.9 (9)	C37—C38—C39—C40	1.5 (11)
C8—C9—C14—C13	175.4 (5)	C38—C39—C40—C41	1.0 (12)
N1—C15—C16—C17	−103.9 (6)	C39—C40—C41—C42	−2.1 (11)
N1—C15—C16—C21	75.1 (6)	C40—C41—C42—C37	0.6 (9)
C21—C16—C17—C18	−1.6 (8)	C38—C37—C42—C41	1.8 (8)
C15—C16—C17—C18	177.5 (5)	C36—C37—C42—C41	176.8 (5)
C16—C17—C18—C19	0.7 (10)	C16—C15—N1—C8	−175.1 (4)
C17—C18—C19—C20	0.1 (12)	C16—C15—N1—C1	61.4 (5)
C18—C19—C20—C21	0.1 (13)	C9—C8—N1—C15	54.6 (6)
C19—C20—C21—C16	−1.0 (11)	C9—C8—N1—C1	179.6 (5)
C17—C16—C21—C20	1.7 (9)	C2—C1—N1—C15	−129.7 (5)
C15—C16—C21—C20	−177.3 (6)	C2—C1—N1—C8	104.2 (5)
N2—C22—C23—C28	85.7 (6)	C37—C36—N2—C22	63.1 (5)
N2—C22—C23—C24	−94.9 (6)	C37—C36—N2—C29	−172.6 (4)
C28—C23—C24—C25	−1.9 (8)	C23—C22—N2—C36	175.0 (4)
C22—C23—C24—C25	178.7 (5)	C23—C22—N2—C29	50.7 (6)
C23—C24—C25—C26	1.5 (9)	C30—C29—N2—C36	54.2 (6)
C24—C25—C26—C27	0.1 (10)	C30—C29—N2—C22	178.8 (4)

### Hydrogen-bond geometry (Å, °)

**Table:**

*D*—H···*A*	*D*—H	H···*A*	*D*···*A*	*D*—H···*A*
N1—H1C···Cl5	0.91	2.19	3.089 (5)	168
N2—H2···Cl5^i^	0.91	2.16	3.066 (4)	172

Symmetry codes: (i) −*x*, −*y*+1, −*z*+1.
